# Human pointing motion during interaction with an autonomous blimp

**DOI:** 10.1038/s41598-022-15016-w

**Published:** 2022-07-06

**Authors:** Mengxue Hou, Qiuyang Tao, Fumin Zhang

**Affiliations:** Georgia Institute of Technology, School of Electrical and Computer Engineering, Atlanta, GA 30308 US

**Keywords:** Electrical and electronic engineering, Mechanical engineering

## Abstract

We investigate the interaction between a human and a miniature autonomous blimp using a wand as pointing device. The wand movement generated by the human is followed by the blimp through a tracking controller. The Vector Integration to Endpoint (VITE) model, previously applied to human–computer interface (HCI), has been applied to model the human generated wand movement when interacting with the blimp. We show that the closed-loop human–blimp dynamics are exponentially stable. Similar to HCI using computer mouse, overshoot motion of the blimp has been observed. The VITE model can be viewed as a special reset controller used by the human to generate wand movements that effectively reduce the overshoot of blimp motion. Moreover, we have observed undershoot motion of the blimp due to its inertia. The asymptotic stability of the human–blimp dynamics is beneficial towards tolerating the undershoot motion of the blimp.

## Introduction

Human pointing motion is one of the major means of indicating intentions. Human pointing devices, such as the computer mouse, are ubiquitous in modern computers. Pointing motion has been leveraged in human–robot interaction (HRI) as a natural and effective way to communicate with robots^1–6^.

Unmanned aerial vehicles (UAVs) are gaining popularity for indoor surveillance, delivery, and warehouse monitoring, where they are required to operate in close proximity to human. In this context, human needs to interact with UAVs in effective ways, and pointing motion should be considered as a preferred method of interaction. Some recent studies have reported promising results on the interaction between human and quadrotors and drones^7–10^ leveraging pointing motions^11,12^. However, these interactions are constrained by safety concerns for the human operator, and there is a lack of mathematical modeling for the pointing device movements generated by the human during the interaction.

The Georgia Tech Miniature Autonomous Blimp (GT-MAB) is a lighter-than-air UAV developed for indoor applications in human-occupied environments. It consists of a saucer-shaped envelope filled with Helium, and a gondola attached to the envelope. The envelope makes GT-MAB naturally cushioned, not posing any safety threat to human. Furthermore, GT-MAB keeps itself aloft without the need for consistent propulsion, due to the buoyancy of the envelope. Therefore, its endurance can be several magnitudes longer than that of a heavier-than-air UAV^13^. Hence GT-MAB is well-suited for carrying out HRI experiments, which often requires the UAV to operate in close proximity to human, and also desires sustained airborne presence to perform repetitive missions^14–18^.

This paper investigates the interactions between a human and the GT-MAB in close proximity, taking advantage of the safety and extended flight time. The human uses a marked wand as the pointing device, which is traced by a localization system. A feedback controller on the GT-MAB achieves tracking of the human pointing motion. Other than our conference paper^19^, we have not found similar investigations of human interaction with robotic blimps using a pointing device in the literature.

Through experimental data collected, we proceed to analyze the wand motion generated by human to control blimp movements. Previous works^11,12^ on human–UAV interaction have not employed mathematical models for human pointing motion. We apply the VITE model, previously applied to model human motion during interaction with a computer mouse^20–22^ to model the wand movements. The experimental data verifies that the VITE model is able to capture the major features in human generated wand movement during the interaction with GT-MAB. On the other hand, our study has shown difference between the motion of the GT-MAB and a computer mouse. Due to inertia, the GT-MAB can not react instantaneously when human changes the direction of wand movement. This has caused the initial response of the blimp movement being away from the target position, which demonstrates a perceivable undershoot in blimp motion. Through theoretical analysis, we can show that the closed-loop dynamics of human–blimp interaction is exponentially stable. The stability indicates that the undershoot blimp motion can be tolerated by the human. Furthermore, the GT-MAB tends to bypass the target position more easily than a mouse cursor displayed on a monitor. We discover that the VITE model may serve as a reset controller used by the human to reduce the overshoot of the blimp motion. This work is a significant extension to our conference paper^19^, where we show preliminary results on modeling human pointing motion with the VITE model, and provide stability analysis. This work presents validation of modeling the pointing motion using the VITE model with experiment data collected by more human subjects. Further, we establish a connection between the reset control method and the VITE model, and discuss the benefits and limitations of the VITE model as a feedback controller with resetting operator, comparing with a linear feedback control law. These discoveries have not been reported in our conference paper^19^, as well as the literature reviewed.

The rest of the paper is organized as follows. “Background: VITE model” section provides a brief introduction on the background of modeling human pointing behavior. The problem formulation is presented in “Problem formulation” section. “Stability analysis” section presents stability analysis of the closed-loop system. Experiment setting and experiment results are shown in “Parameter identification and validation” section, and the discussion on human behavior analysis is presented in “Human behavior analysis” section. “Conclusion” section describes the conclusion of the paper based on the experiment results.

## Background: VITE model

The VITE model is a second order dynamic model for human pointing motion^20^. Consider a human controls the position of a pointing device, such as a computer mouse, which displays a visible pointer such as a cursor on a monitor. We assume the human intends to move the pointer to a desired location. Let *y*(*t*) represent the position of the pointing device under human control. Let *u*(*t*) represent the perceived position of the pointer, $r_t$ denote the desired position of the pointer, then $r_t - u(t)$ represents a difference vector describing the difference between the displayed pointer position and the desired position. The VITE model describes the motion of the pointing device as follows:

1 $$\begin{aligned} \left\{ \begin{array}{ll} \dot{\eta }(t) = \gamma (-\eta (t) + r_t -u(t))\\ \dot{y}(t) = g[\eta (t)]^{+}_{d} \end{array}\right. , \end{aligned}$$

where $\eta (t)$ represents an internal state describing how the human perceives the difference vector, which cumulatively integrates the difference vectors over time with a constant gain $\gamma$. The operator $[\cdot ]^{+}_{d}$ is used to switch the pointing motion off when the displayed pointer bypass its target. It is defined by the following equation

2 $$\begin{aligned}{}[v]^{+}_{d}= \left\{ \begin{array}{ll} v, &{\quad} {\text{if}} \langle v,d \rangle \ge 0 \\ 0, &{\quad} {\text{otherwise}} \end{array}\right. , \end{aligned}$$

where *d* defines the direction from the pointer to the target position at initial time *t* = 0. The parameter *g* is called the go signal, which is a feedback gain describing how the internal state $\eta (t)$ results in the pointer motion *y*(*t*). The signature property of the VITE model is that the displayed pointer tends to move beyond the target, generating an overshoot. This has been confirmed to agree with human behaviors by experimental work on human pointing motion^22,23^.

The pointing task is modeled as a feedback control that generates the wand position as the input to the blimp system. The blimp position is the output that will be controlled to a desired position. This is considered as an output regulation problem for controller design. The VITE model is viewed as a feedback control law that produces the wand position *y*(*t*) based on the difference vector $r(t)-u(t)$. In the context of human computer interface using a computer mouse, the human drives the displayed cursor position *u*(*t*) to the target position $r_t$, by controlling the position of the mouse *y*(*t*). We notice that the VITE model bears similarity with the reset controller in control theory literature^24,25^. Comparing to regular feedback controllers, a reset controller typically defines a reset condition. At the time that the reset condition is met, the control effort is set to zero. The control effort of the reset controller switches back to non-zero values if the reset condition is not met. Due to the switching condition in (2), the VITE model can be viewed as a variant of the reset controller. However, the human considers the pointing task fulfilled when the reset condition is satisfied, and the control effort *y*(*t*) stays at zero for all time after the reset condition is met. It is known that a reset controller might reduce overshoot in the step response of a feedback control system^26^. However, such effect has not been reported in human robot interaction through pointing motion.

## Problem formulation

**Figure 1:**
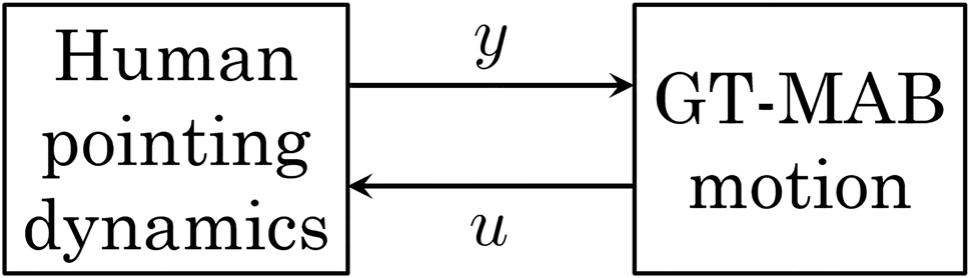
Block diagram of the closed-loop dynamics. Human generates wand movement *y*(*t*) while observing blimp movement *u*(*t*).

We investigate human pointing motion when interacting with the GT-MAB. as illustrated by Fig. 1. The human operator observes blimp position and then moves a marked wand with its position followed by the GT-MAB. The human will control the blimp towards a target position that is unknown to the blimp. We perform a series of pointing experiments where data on the wand movements and blimp movements are collected.

To simplify the dynamics model, we introduce the Assumption 1 in^19^, that the horizontal position of the target that the human specifies is the same as the horizontal position of the blimp. Due to this Assumption, we use *y*(*t*) to represent the vertical location of the wand, *u*(*t*) to represent the vertical position of the blimp, and *r*(*t*) to represent the vertical location of the target. We will then attempt to model the human pointing motion using the VITE model.

### Assumption 1

We assume that when interacting with the blimp, the wand movements can be modeled by (1).

### Remark 1

It has been shown that human exhibits similar motions when reaching and pointing with their arms, mouse pointers and other devices^27^. VITE model has been adopted for modeling human pointing motion in many existing work on HCI, e.g.^21^. Hence we assume that the human blimp interaction behavior can be described by the VITE model as well. Experimental justification of this assumption is provided in Section Parameter identification and validation.

From our previous work^14^, the vertical motion of GT-MAB can be described as

3 $$\begin{aligned} \begin{aligned} m \ddot{u}(t) + F_z(t) = f_z(t), \end{aligned} \end{aligned}$$

where $m = m_{RB}+m_{Az}$, $m_{RB}$ and $m_{Az}$ are the rigid-body mass of the blimp and the added mass for the vertical motion. The term $F_z(t) = D \dot{u}(t)$ where *D* is the aerodynamic drag coefficient for the vertical movement. As justified in our previous paper^28^, *D* can be well approximated as a positive constant for indoor miniature blimps. The term $f_z(t)$ represents the thrust force in the vertical direction.

Feedback controllers are implemented to control the position and heading of the blimp. For convenience, GT-MAB keeps the same heading angle through the entire experiment, and the reference setpoint for horizontal movement is set to a fixed position, so that it will remain at the same horizontal position throughout the experiment. Setpoint of the vertical motion is set to the height of the wand, thus enabling the blimp to track position of the wand. The height controller can be described as $$ f_z(t) = k_p e(t), $$ where *e*(*t*) = *y*(*t*) − *u*(*t*) represents the difference between the height of the blimp and the height of the wand. $$k_p> 0$$ denotes the feedback gain. The blimp dynamics and the human pointing dynamics are connected to form a closed-loop feedback system as shown in Fig. 1. The first objective of this paper is formulated as the following problem:

### Problem 1

Prove that the closed-loop system formed by the human pointing dynamics and the blimp dynamics is asymptotically stable.

Concerns on stability might not be of high priority for human computer interface because mouse movement is agile. For the GT-MAB, however, due to inertia and air drags, the bimp might not react instantaneously to wand movement. A theoretical justification of the asymptotic stability of the closed-loop system will ensure that the human–blimp interaction experiments can be carried with meaningful and predictable results.

After collecting the wand movement data from the experiments, we will identify the parameters of the VITE model for each human subject. The parameters of the VITE model include the unknown gains $g, \gamma$, and the unknown reference target positions $r_t$. The problem is formulated as follows:

### Problem 2

Given a set of wand trajectories, denoted as *y*(*t*) collected from multiple experiments, identify the unknown VITE parameters $g, \gamma$, and the unknown reference signal $r_t$.

Solution to this problem will also be used to justify that the VITE model can capture the features of human pointing motion in the context of human–blimp interaction.

We have noticed that the VITE model can be viewed as a reset controller used by the human to reduce the difference between the blimp position and the target position. Given many possible feedback controllers that are able to achieve the same goal, we are interested to know why the VITE model was adopted by human. Specifically, we formulate the following problem to compare the VITE model with a linear feedback law:

### Problem 3

Identify the benefits and limitations of the VITE model as a reset controller in the context of human blimp interaction, comparing to a linear feedback controller without the reset operator.

## Stability analysis

In this section, we show that the closed-loop dynamics formed by the VITE model and the blimp dynamics are asymptotically stable. We assume that the target position is located at the origin, and blimp’s initial position is lower than the target position. Detailed description and justification of this assumption is provided in^19^.

Given the blimp dynamics described in (3), and the human pointing motion dynamics as in (1), we introduce an augmented state $\mathbf {x} \in \mathbb {R}^4$, where elements of $\mathbf {x}$ are defined as $$\begin{bmatrix} x_1&x_2&x_3&x_4 \end{bmatrix}^T \triangleq \begin{bmatrix} u&\eta&\dot{u}&y \end{bmatrix}^T,$$ then the closed-loop system dynamics can be written as

4 $$\begin{aligned} \begin{aligned} \dot{\mathbf {x}}&= \begin{bmatrix} x_3 \\ -\gamma (x_1+x_2) \\ - \frac{D}{m} x_3 +\frac{k_p}{m} (x_4-x_1)\\ g x_2 ^+\\ \end{bmatrix}, \end{aligned} \end{aligned}$$

where $x_2^+$ denotes the non-negative portion of $x_2$, that is, $x_2^+ = x_2$ when $x_2 \ge 0$, and $x_2 = 0$ when $x_2 < 0$. The closed loop dynamics has the following equilibrium set: $E = \{ x_3 = 0, \hbox{ and } \, x_1 = x_4 = -x_2, \hbox{ and } \, x_2 \le 0\}.$

We will now examine the stability of the closed-loop system dynamics in (4) considering two cases, the first case where $x_2 \ge 0$, and the second case $x_2 <0$. In both cases, the system dynamics is linear, while a switch in system dynamics happens when $x_2$ goes from $x_2 > 0$ to $x_2 < 0$. Define a vector $\mathbf {z}\in \mathbb {R}^4$ as $$\mathbf {z} = \begin{bmatrix} x_1 - x_4&x_1 + x_2&x_3&x_2^+ \end{bmatrix}^T.$$ In the case where $x_2 \ge 0$, the dynamics for $\mathbf {z}$ is

$$\begin{aligned} \dot{\mathbf {z}} = \begin{bmatrix} 0 &{} 0 &{} 1 &{} -g \\ 0 &{} -\gamma &{} 1 &{} 0\\ -\frac{k_p}{m} &{} 0 &{} -\frac{D}{m} &{} 0\\ 0 &{} -\gamma &{} 0 &{} 0 \\ \end{bmatrix} \mathbf {z} := \mathbf {A}_1 \mathbf {z}. \end{aligned}$$

In the case where $x_2 < 0$, $z_4 = x_2^+ = 0$. Thus, $\dot{z}_4 = 0$, and the dynamics of $\mathbf {z}$ can be described as

$$\begin{aligned} \dot{\mathbf {z}} = \begin{bmatrix} 0 &{} 0 &{} 1 &{} 0 \\ 0 &{} -\gamma &{} 1 &{} 0 \\ -\frac{k_p}{m} &{} 0 &{} -\frac{D}{m} &{} 0\\ 0 &{} 0&{} 0 &{} 0 \\ \end{bmatrix} \mathbf {z} := \mathbf {A}_2 \mathbf {z}.\end{aligned}$$

For the first case, the following Lemma holds.

### Lemma 1

The closed-loop system $\dot{\mathbf {z}} = \mathbf {A}_1 \mathbf {z}$ is exponentially stable if $k_p > \frac{g(D + \gamma m)(\gamma ^2 + D) - \gamma D^2 - m\gamma ^2 D}{D}$.

### Proof

We will prove the above lemma using Routh’s stability criterion. The characteristic polynomial is

5 $$\begin{aligned} \begin{aligned} \det (\lambda I - \mathbf {A}_1)&= \lambda ^4 + \left(\frac{D}{m} + \gamma \right)\lambda ^3 + \left(\gamma \frac{D}{m} + \frac{k_p}{m}\right)\lambda ^2 + \frac{\gamma k_p}{m} \lambda + \frac{g k_p \gamma }{m}. \end{aligned} \end{aligned}$$

The matrix $\mathbf {A}_1$ is Hurwitz if the first column of the Routh array is positive. Denote the first column of the Routh array as $a_0, a_1, b_1, c_1, d_1$. $a_0, a_1, b_1$ and $d_1$ are guaranteed to be positive given any choice of $D, \gamma , g, k_p$. If $k_p > \frac{g(D + \gamma m)(\gamma ^2 + D) - \gamma ^2 - m\gamma ^2 D}{D}$, then $c_1>0$ and the system is stable by the Routh stability criteria. $\square$

Now consider the second case where $x_2<0$. In this case, $z_4$ has already converge to zero, we will consider the stability of the subsystem $\dot{\tilde{\mathbf {z}}} = \tilde{\mathbf {A}}_2 \tilde{\mathbf {z}}$, where $\tilde{\mathbf {z}} = [z_1, z_2, z_3]^T$, $\tilde{\mathbf {A}}_2$ is the third order leading principal submatrix of $\mathbf {A}_2$.

### Lemma 2

The subsystem $\dot{\tilde{\mathbf {z}}} = \tilde{\mathbf {A}}_2 \tilde{\mathbf {z}}$ is exponentially stable for all $H, g, \gamma , k_p >0$.

### Proof

The characteristic polynomial is $\det (\lambda I - \mathbf {A}_2) = \lambda ^3 + (\frac{D}{m} + \gamma )\lambda ^2 + \frac{D \gamma + k_p}{m}\lambda +\frac{ k_p \gamma }{m}.$ For all $D_{\omega z}, g, \gamma , k_p >0$, the first column of the Routh array is positive. Therefore, the closed-loop system is exponentially stable. $\square$

The above two Lemmas lead to the following Theorem. Detailed proof can be found in^19^.

### Theorem 1

For the system dynamics (4), with $k_p > \frac{g(D + \gamma m)(\gamma ^2 + D) - \gamma D^2 - m\gamma ^2 D}{D}$, if the states start from $x_2 > 0$, the states will exponentially converge to the equilibrium set *E*.

## Parameter identification and validation

In this section, we describe the experiment setting for human blimp interaction, and process the experimental data to justify Assumption 1 and to identify the unknown parameters of the VITE model. The stability of the closed-loop system is verified wit the parameters identified from the experimental data.

### Experiment setting and data processing

**Participants** Five unpaid participants (2 female, mean age 24 years old) are recruited for the study, all with normal eyesight, and all practiced to be familiar with GT-MAB dynamics.

**Task** We asked the participant to determine two target positions at different heights at their will, and then drive a GT-MAB from its initial position to the first target position by moving the wand. Once the participant is satisfied with the blimp position, he/she will change the direction of wand movement and drive the blimp towards the second target position. The participant was asked to keep repeating this task for 60 s. One video of the experiment can be found at https://youtu.be/4JavPaOVKio. Note that in some of the experiments, Assumption 1 in^19^ is violated. Due to the wind disturbance, the blimp may have horizontal displacement, even though a horizontal station keep feedback control law is implemented.

**Facilities** The experiment took place in a space that is 8 m long, 7.5 m wide, and 3.5 m high. Flex13 cameras from OptiTrack are installed on the walls, at about 3.5 m height. Reflective markers are attached on the top of GT-MAB envelope as well as on the tip of the wand, so that the OptiTrack system can detect and record position of the wand and the blimp. The OptiTrack system captures the trajectory of the blimp and the wand at 10 Hz sampling frequency. Control commands are sent to the GT-MAB via wireless communication enabled by the XBee device. The blimp controller is implemented using MATLAB at 10 Hz, on a Core i-7 2.93 GHz PC with 16GB RAM. The blimp and wand trajectories are logged as they are captured by the OptiTrack system.

**Figure 2:**
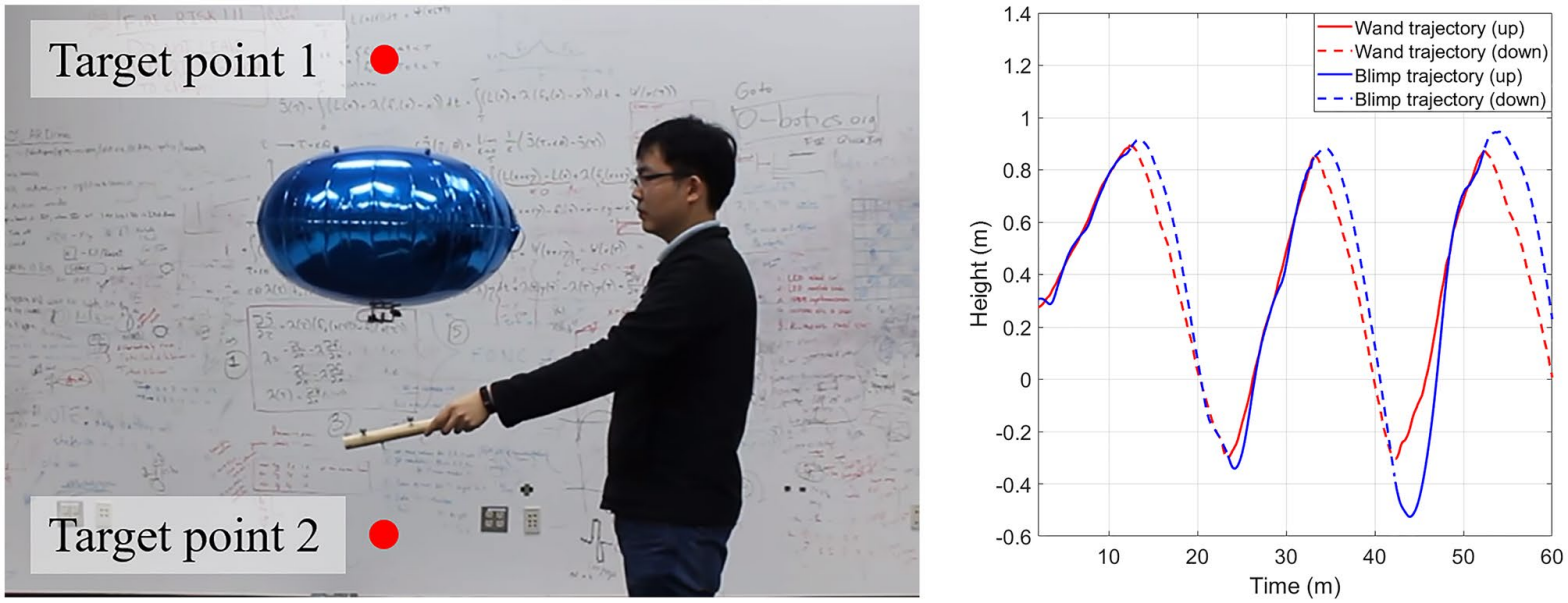
(**Left**): Demonstration of experiment settings. OptiTrack camera system is installed on the walls to measure GT-MAB and wand trajectories. The horizontal position of human is about 1 m away from the horizontal position of GT-MAB. (**Right**): Blimp trajectory and wand trajectories collected from the experiment.

Considering the limited coverage of OptiTrack cameras, the human and the blimp will stay inside a 4 m long, 4 m wide, and 2 m high space during the experiment. The starting position of blimp is about 1 m away from the human, and is about 1.5 m high, while the horizontal target position of the blimp will be the same as the starting position, and the vertical target position will be between 0.5 m and 2 m. Figure 2 (Left) shows a demonstration of the experiment settings.

**Data preprocessing** It has been shown that when human performs the pointing motion, the pointer velocity data is noisy^29^. To address this issue, we filter the wand velocity using a Savitzky–Golay filter with a 4th order polynomial and a window size of 81 (8 s), and calculate the smoothed wand trajectory by integrating the filtered velocity. The smoothed wand trajectory, denoted as $\tilde{y}(t)$, is used as the input data for solving the parameter identification problem.

We divide the smoothed wand trajectory $\tilde{y}(t)$ into several going-up and going-down segments, according to the movement of the wand. Since the trial is considered to be finished once the human stops moving the wand, indicating that the human is satisfied with the blimp height, the time interval of the experiment can be divided into $[T_1^-, T_1^+]$, $[T_2^-, T_2^+], \ldots , [T_{N}^-, T_N^+]$ based on the up/down motion of the wand, where $T_{n}^-$ and $T_{n}^+$ denote the starting and ending time of the *n*th trial, $T_{n-1}^+ = T_{n}^-$. The ending time of each trial is the timestep when the wand stops moving, $\dot{\tilde{y}}(t)=0$. In each set of experiment data, we delete the data points that do not belong to a complete trial. Figure 2 (Right) shows one set of blimp and wand trajectories divided into up/down sections.

**Parameter estimation**

Let $\Omega _{u}=\{n\in \mathbb {Z}|\dot{\tilde{y}}(t) > 0, t\in [T_{n}^-, T_n^+]\}$ represent the set of all going-up sections. Similarly, let $\Omega _{d}=\{n\in \mathbb {Z}|\dot{\tilde{y}}(t) < 0, t\in [T_{n}^-, T_n^+]\}$ denote the set of going-down sections. Let $r_{t,u}$ and $r_{t,d}$ denote the target position of all the going-up sections and going-down sections respectively. We represent the set of unknown parameters in the VITE model as ${\Phi } = [\hat{g}, \hat{\gamma },\hat{r}_{t,u}, \hat{r}_{t, d}]$. Given a set of parameters $\Phi$ as well as the actual blimp trajectory *u*(*t*) from the experiment, a simulated wand trajectory $\hat{y}(t)$ can be derived from the following initial value problem, where the wand trajectory is simulated by the VITE model. Further, at the start and the end of each segment, since the human stops moving the wand, the human internal state must be zero.

6 $$\begin{aligned} \begin{aligned} \dot{\hat{\eta }}(t)&= \left\{ \begin{array}{c l} \hat{\gamma }(-\hat{\eta }(t) + \hat{r}_{t,u} - u(t)),\quad {\text {if}}\, n\in \Omega _{u}\\ \hat{\gamma }(-\hat{\eta }(t) + \hat{r}_{t,d} - u(t)), \quad {\text{if}}\, n\in \Omega _{d}\\ \end{array}\right. ,\\ \dot{\hat{y}}(t)&= \hat{g}[\hat{\eta }(t)]_d^{+}, \\ \hat{y}(0)&= \tilde{y}(0), \\ \hat{\eta }(T_{n}^-)&= 0, n\in \Omega _{\rm u} \cup \Omega _{\rm d}. \end{aligned} \end{aligned}$$

Hence we formulate the parameter identification problem as a constrained optimization problem. The optimization problem is minimizing the difference between the simulated and the smoothed wand wand trajectory. To solve the optimization problem, we convert the terminal constraint that the human internal state is zero to a penalty term in the cost function, as follows:

7 $$\begin{aligned} \begin{aligned}{}&\min _{{\Phi }} \sum _{n\in \Omega _{u}}{\int _{T_{n}^-}^{T_n^+}{(\tilde{y}(t) - \hat{y}(t))^2 d t}} + \sum _{n\in \Omega _{d}}{\int _{T_{n}^-}^{T_n^+}{(\tilde{y}(t) - \hat{y}(t))^2 d t}} + \beta \sum _{n\in \Omega _{u}\cup \Omega _{d}} \hat{\eta }(T_n^+)^2 \\&{\text {s.t. }} \dot{\hat{\eta }}(t) = \left\{ \begin{array}{ll} \hat{\gamma }(-\hat{\eta }(t) + \hat{r}_{t,u} - u(t)), &\quad {\text {if}}\, n\in \Omega _{u}\\ \hat{\gamma }(-\hat{\eta }(t) + \hat{r}_{t,d} - u(t)) &\quad {\text {if}}\, n\in \Omega _{d}\\ \end{array}\right. ,\\&\quad \ \dot{\hat{y}}(t) = \hat{g}[\hat{\eta }(t)]_d^{+}, \\&\quad \ \hat{y}(0) = \tilde{y}(0), \\&\quad \ \hat{\eta }(T_{n}^-) = 0, n\in \Omega _{\rm u} \cup \Omega _{\rm d}, \end{aligned} \end{aligned}$$

where $\beta \in \mathbb {R}$ is a parameter chosen to balance the penalty term and the modeling error. We set $\beta = 50$, and solve the above optimization problem using the interior point method^30^. Since the cost function is non-convex with respect to the parameters, we apply the global optimization technique^31^ to avoid the solution converging to a local minimum.

The identified parameters are given in Table 1. We identify the VITE model parameters $\hat{\gamma }, \hat{g}$ for both participants. The two participants perform the pointing motion with different model parameters. Further, the identified parameters of each of the participants in different trials show consistency, which is indicated by the small variance of the identified parameters in different experiments.

**Table 1:** Identified parameters and RMSE between the actual and simulated wand trajectory.

		$\hat{\gamma }$	$\hat{g}$	traj $\hbox{RMSE}_{\rm m}$	traj $\hbox{Var}_{\rm m}$	traj $\hbox{RMSE}_{\rm t}$	traj $\hbox{Var}_{\rm t}$	vel $\hbox{RMSE}_{\rm m}$	vel $\hbox{RMSE}_{\rm t}$
S1	D1	0.4010	0.2333	0.1057	0.0131	0.2228	0.0121	0.0100	0.0241
	D2	0.6584	0.2189	0.0571	0.0020	0.0576	0.0026	$1.376e^{-4}$	0.0083
	D3	0.5318	0.1768	0.0912	0.0038	0.1421	0.0062	0.0104	0.0363
	Mean	0.5304	0.2097	0.0847	–	0.1408	–	0.0068	0.0229
	Var.	0.0166	$8.62e^{-4}$	–	–	–	–	–	–
S2	D4	0.3973	0.3618	0.0241	$5.7729e^{-4}$	0.0280	$7.7702e^{-4}$	0.0030	0.0104
	D5	0.4341	0.2906	0.0615	0.0019	0.0731	0.0037	0.0067	0.0095
	D6	0.4208	0.2371	0.0426	0.0015	0.0465	0.0023	0.0039	0.0070
	Mean	0.4174	0.2965	0.0543	–	0.0492	–	0.0045	0.0090
	Var.	$3.4723e^{-4}$	0.0039	–	–	–	–	–	–
S3	D7	0.2784	0.2001	0.0937	0.0084	0.0876	0.0074	0.0024	0.0387
	D8	0.3102	0.2456	0.0743	0.0036	0.0906	$9.6341e^{-4}$	0.0031	0.0009
	D9	0.3031	0.2093	0.0575	0.0032	0.0720	0.0043	0.0035	0.0042
	Mean	0.2972	0.2183	0.0752	–	0.0834	–	0.0030	0.0158
	Var.	$$ 2.78e^{-4}$$	$$5.78e^{-4}$$	–	–	–	–	–	–
S4	D10	0.3711	0.3183	0.1074	0.0113	0.1387	0.0045	0.0040	0.0107
	D11	0.8962	0.6536	0.1118	0.0103	0.2231	0.0633	0.0193	0.0463
	D12	0.4012	0.2466	0.1491	0.0041	0.0928	0.0087	0.0092	0.0085
	Mean	0.5562	0.4062	0.1228	–	0.1515	–	0.0108	0.0218
	Var.	0.0869	0.0472	–	–	–	–	–	–
S5	D13	1.5950	0.2087	0.0755	0.0046	0.0801	0.0023	0.0024	0.0031
	D14	1.2490	0.2949	0.0471	0.0020	0.1533	0.0038	0.0049	0.0135
	D15	1.1645	0.2341	0.0678	0.0040	0.0774	0.0021	0.0022	0.0109
	Mean	1.3362	0.2459	0.0635	–	0.1036	–	0.0032	0.0092
	Var.	0.0520	0.0020	–	–	–	–	–	–

S1–S5 represent the two human participants, while D1–D15 are the 15 datasets collected in the experiments. traj $\hbox{RMSE}_m$ shows the RMSE between the true and the simulated wand trajectory in the training set, and traj $\hbox{RMSE}_t$ presents the RMSE in the test set (in m). traj $\hbox{Var}_m$ and traj $\hbox{Var}_t$ are the variance of the difference between the true and the simulated wand trajectory, for the training set and the test set, respectively. Similarly, vel $\hbox{RMSE}_m$ and vel $\hbox{RMSE}_t$ shows the RMSE between the true and the simulated wand velocity, in the training set and the test set, respectively (in m/s).

### Justification of Assumption 1

To verify whether the VITE model is applicable to human blimp interaction, we divide the up/down sections into two sets, the training set and the testing set. The training set is the set of complete going-up and going-down segments in the first 35 s, while the test set contains the rest of the trajectory. We first compare the smoothed wand trajectory in experiment with the wand trajectory simulated by the VITE model with the identified parameters. Since the VITE model is a second order dynamical system, we also compare the smoothed wand velocity in experiment with the VITE model simulated wand velocity.

**Figure 3:**
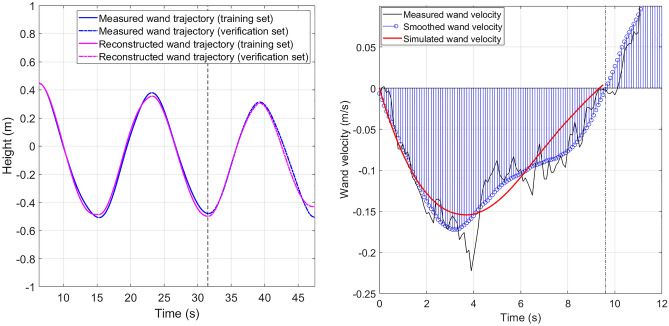
(**Left**): Comparison between the measured and the simulated wand trajectory. (**Right**): Comparison between the measured wand velocity computed from differentiating the trajectory data, the smoothed wand velocity, and the simulated velocity (dataset 5).

**Justification using the wand position data** The Root Mean Square Error (RMSE) between the true and the simulated wand trajectory and its variance is given in Table 1. For the two participants, the RMSE for the training set is less than 7.2% of the total change-of-height of the blimp throughout the experiment. The RMSE of the test set is larger than the training set, and is about 11.6% of the total change-of-height of the blimp. The small RMSE and the low variance indicate that the VITE model can accurately describe the human blimp interaction, and it can correctly predicts the human behavior. Figure 3 (Left) shows one set of comparison between the measured and the reconstructed wand trajectory. It can be seen that the two trajectories match well in both the training and the test set. This indicates that the VITE model simulated trajectory is able to describe and predict the human pointing behavior in human blimp interaction.

**Justification using the wand velocity data** We also provide justification of the VITE model by computing the RMSE between the true and simulated wand velocity. The RMSE is given in Table 1. The RMSE for the training set is less than 8.5% of the maximum wand velocity throughout the experiment, while the RMSE is less than 15.2% of the maximum wand velocity in the test set. Figure 3 (Right) shows one set of the measured, smoothed and simulated wand velocity, where the simulated wand velocity is computed by $\hat{g}\eta (t)$. It can be seen that the simulated wand velocity matches with the smoothed wand velocity, which shows that the VITE model can qualitatively reproduce the wand velocity. The VITE model matches with the wand velocity better at the beginning of each segment, in the acceleration phase. There is relatively larger error when the blimp is about to reach the target position, and the human decelerates the wand. One possible explanation for the modeling error in the deceleration phase is that in the experiment, human is not given a specific target position. Hence the participant does not pay attention to the accuracy of the pointing motion, and tends to drive the blimp towards the target with higher speed than the VITE model predicts.

From the error analysis, we observe that the human blimp interaction can be described and predicted by the VITE model. This is an expected outcome since the VITE model describes a general relationship between the distance from the target to the pointer and the neural commands of muscles contraction for human pointing motion in different interfaces.

## Human behavior analysis

In this section, we describe human and blimp behaviors during the experiments. We will compare these behaviors with the behaivors observed in human computer interface experiments. In order to describe observations of the experiment, we use the following visualization techniques in existing literature on HCI:

**Time-series plots** We plot the wand position and velocity over time, to describe the human behavior in time.

**Phase space plots** The phase space trajectory describes the evolution of the system state in the VITE model with respect to the system input, which is the blimp position.

###  Wand and blimp movements

Figure 2 (Right) shows one set of experimental data. We observe that the human moves the wand towards the target with accelerated motion at the beginning. As the blimp goes near the target position, the human slows down the pointing motion, until he/she is satisfied with the blimp position. After the human stops moving the wand, the blimp slows down, and keeps moving up/down for a short period of time before its speed reduces to zero.

**Figure 4:**
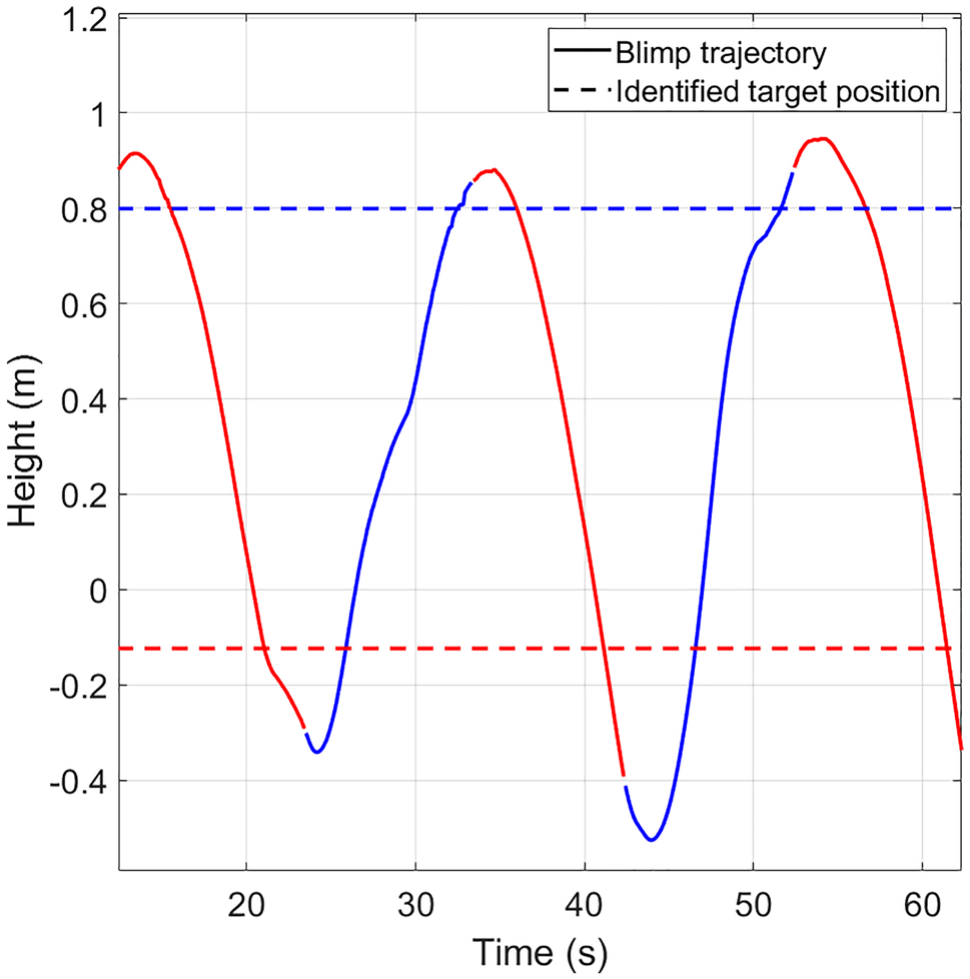
Comparison between the identified target position and the blimp trajectory. The blue and red solid lines show the blimp trajectory in the going-up/down segments, respectively, and the blue and red dashed lines show the identified target position for the going-up/down segments.

As shown in Fig. 4, comparing to the identified target position with the blimp trajectory, the stopping position of the blimp in each going up/down segment goes over the identified target position. This overshoot can be explained by the VITE model. Let’s take one going-up segment as an example. Suppose the internal state has not reached zero, then the human internal state can be described by integrating the system input,

8 $$\begin{aligned} \eta (t) = \int \limits _{0}^t \gamma \exp (-\gamma (t-\tau )) (r_t - u(\tau ))d\tau . \end{aligned}$$

Suppose at time step $t'$, $u(t')$ reaches $r_t$. This is the timestep that the input to (8) goes to zero, meaning that the human stops accelerating the wand. However, at this timestep, $\dot{y}(t') = g [\eta (t')]_d^+>0$, since the human acceleration is positive, $r_t - u(t)>0, \forall t\in [0,t')$. Hence the human does not stop the wand movements when the blimp reaches the target position. Instead, when human stops moving the wand, the blimp has already moved over the target position. This explains the overshoot observed in Fig. 4.

**Figure 5:**
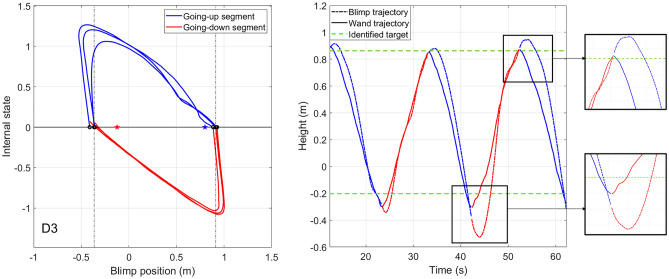
(**Left**): The internal state trajectory (dataset 3). Black circles show the initial state of the system. The blue and red straight lines are the equilibrium set of the going-up/down segments. The identified target position of the going-up/down segments is shown by the blue/red star. (**Right**): Blimp and wand trajectory. As shown in the zoomed-in plot, after the human stops moving the wand and switch the target position, the blimp takes additional time to change its direction. This results in the “wrong”initial response of the closed-loop system.

Moreover, we observe an “undershoot”in the blimp trajectory. Figure 5 (Left) shows the trajectories of the closed-loop pointing system. The human internal state is plotted against the blimp position. From the plot, It can be seen that the blimp first move in the opposite direction of the target position, i.e., in the going-up segments, the blimp first goes down before going up towards the equilibrium set. The system state first moves in the “wrong”initial direction, but then it eventually reverses course and reaches the desired steady state. As shown in Fig. 5 (Right), since the blimp is a second-order system, direction change will take more time than a computer mouse. Hence, after the human stops the wand and switches to the next target, the blimp takes additional time to reduce its speed to zero, before changing its direction to go to the next target. At the initial time of each going-up/down segment, the initial condition of the blimp is not zero. The wrong initial direction of the blimp is the natural response of the blimp driven by the system’s initial states. Since the closed-loop system is exponentially stable, the blimp catches up with the wand trajectory after a short period of time, as shown in Fig. 5 (Right).

The initial response in the “wrong”direction is an undesirable effect in practice. In human blimp interaction experiments, the user might get confused whether the blimp has finished reaching the previous target, and may suspect that the blimp is malfunctioning. In such cases, incorporating feedback from the blimp in reaction to the human user may provide better interaction experience^18^.

### Benefit and limitation of the VITE model

We refer to the VITE model without the reset term as the baseline controller, $\ddot{y}(t) = g\gamma (-\eta (t) + r_t -u(t)).$ It is easy to prove that the equilibrium of the closed-loop system formed by the base controller and the blimp is the origin. When the blimp reaches the target position, we have $u = r_t$. In this section, we aim to identify the benefits and limitations of the VITE model by comparing it against the baseline controller.

**Figure 6:**
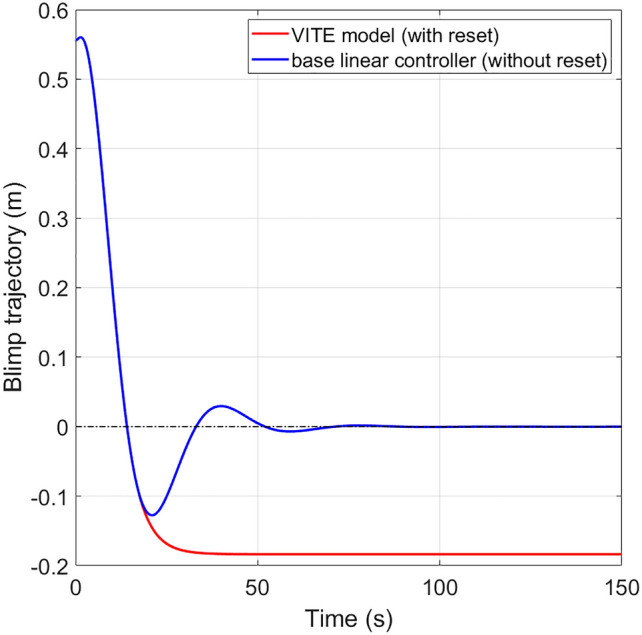
Simulated blimp trajectory under the base controller without the reset operation, and the VITE model. The initial position and velocity of the blimp are identical from the experiment, and the parameters of the VITE model are identified from dataset 1.

Compared with the baseline controller, the major benefit of the VITE model is that it reduces the overshoot of blimp motion. Figure 6 shows comparison between the tracking error of using the VITE model and the baseline controller to track a fixed target position. Under the VITE control, the blimp motion settles at the equilibrium much faster than the baseline controller, with significantly less overshoot. It has been theoretically justified that the reset controller reduces overshoot and settling time of a system under control^25^.

However, the equilibrium of the closed-loop system formed by the VITE model and the blimp is not the origin. Once the reset condition $\eta = 0$ is satisfied, the human perceives the task finished, and stops wand movement. Therefore, the VITE model will not drive the tracking error to zero. On the other hand, the baseline controller will drive the blimp position to the origin, achieving better accuracy than using the VITE model.

The use of the reset controller indicates that when interacting with the blimp, human prefers to fulfill the task in shorter time with less overshoot while sacrificing accuracy. This phenomenon is also seen in pointing motion across various interfaces, i.e. HCI using a mouse^29^. The human adopts a two-phase mechanism to fulfill the pointing motion, the surge phase and the corrective phase. The surge phase denotes the initial movement towards the target. In this phase human points in an accelerated motion. After the pointer is adjacent to the target, human uses a slower corrective motion to let the tracking error reach zero^32^.

## Conclusion

We investigate interaction between a human user and an autonomous blimp by letting the human control the position of the blimp through wand movements. We verify that the VITE model can describe human generated wand motion when interacting with the blimp. We show that the closed-loop system describing the human–blimp interaction is exponentially stable. The exponential stability tolerates the undershoot behavior of the blimp caused by its inertia. Moreover, the study suggests that the VITE model, as a special reset controller, reduces the overshoot of the blimp motion in human–blimp interaction.

### Ethics approval

This study was performed with approval granted by office of research integrity assurance, Georgia Institute of Technology. All experiments were performed in accordance with relevant guidelines and regulations.

### Consent to participate

Informed consent was obtained from all individual participants included in the study.

### Consent to publish

The authors affirm that human research participants provided informed consent for publication of Figs. 1, 2(Left), 3, 5, and video https://www.youtube.com/watch?v=4JavPaOVKio.

## Supplementary Information

Supplementary Information.

## Data Availability

The datasets generated during and/or analysed during the current study are available in the human–blimp-interaction repository, https://github.com/mengxueHou/.
