# Supplementary material for: Human pointing motion during interaction with an autonomous blimp
Source: Sci Rep. 2022 Jul 6;12:11402. doi: 10.1038/s41598-022-15016-w (PMC9259637; doi:10.1038/s41598-022-15016-w)
Supplement: Supplementary file 1 — Supplementary Information. [file 41598_2022_15016_MOESM1_ESM.pdf]

Supplementary material

We provide visualization of the full dataset in this supplementary material. Figure 1 shows the comparison between the simulated wand trajectory and the actual wand trajectory. The statistics of the difference between the simulated and actual wand trajectory is in Table 1.

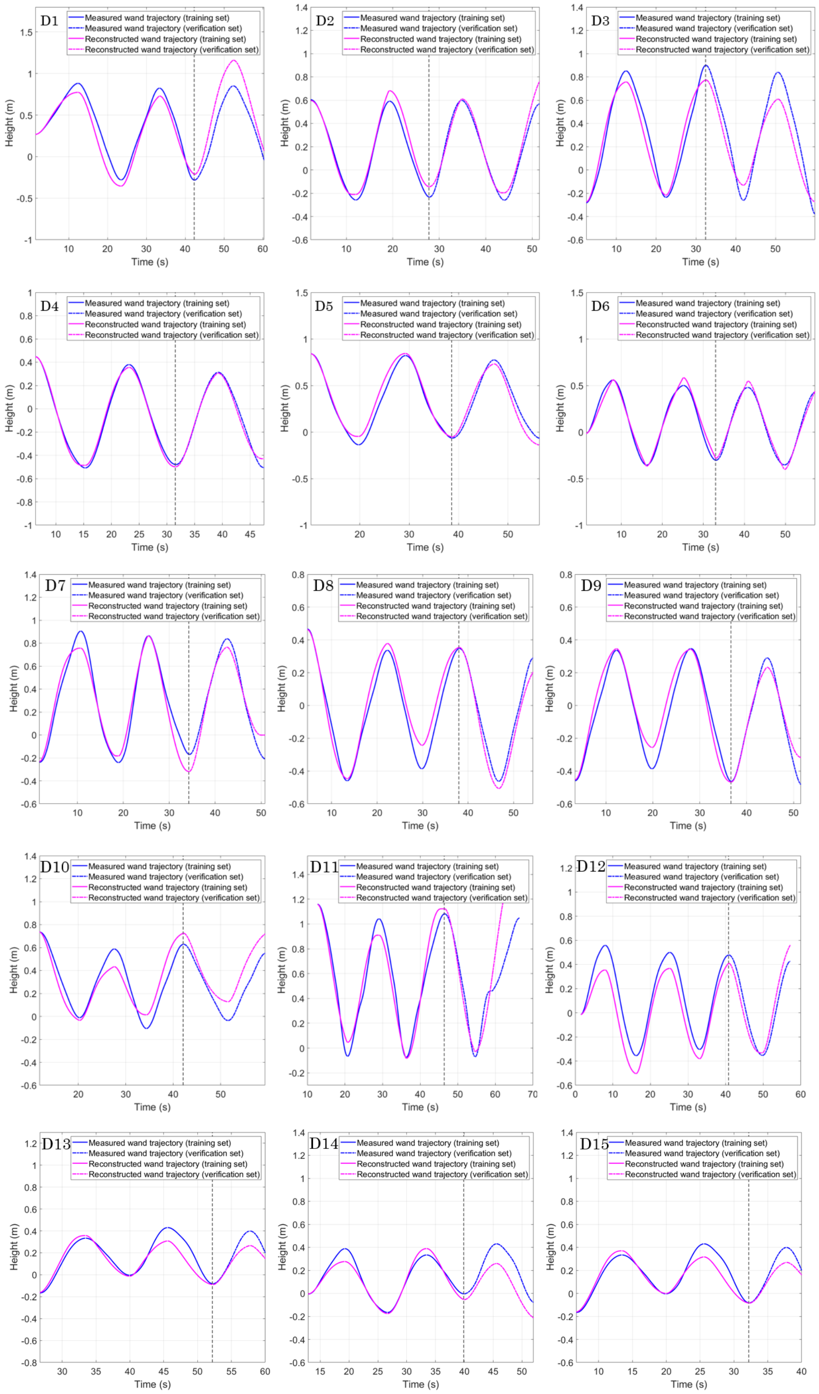

Fig. 1. Comparison between the measured and the simulated wand trajectory (all dataset).
